# Postgraduate Year Two Medical Residents’ Awareness of Personal Development as a Physician during the Management of Inpatients: A Qualitative Study

**DOI:** 10.3390/healthcare12161621

**Published:** 2024-08-14

**Authors:** Kazuki Tokumasu, Haruo Obara, Takanobu Hirosawa, Hiroko Ogawa, Fumio Otsuka

**Affiliations:** 1 Department of General Medicine, Okayama University Graduate School of Medicine, Dentistry and Pharmaceutical Sciences, Okayama 700-8558, Japan; 2 Department of General Internal Medicine, Okinawa Chubu Hospital, Uruma 904-2243, Japan; 3 Department of Diagnostic and Generalist Medicine, Dokkyo Medical University, Shimotsuga, Tochigi 321-0293, Japan; 4 Department of Primary Care and Medical Education, Okayama University Graduate School of Medicine, Dentistry and Pharmaceutical Sciences, Okayama 700-8558, Japan

**Keywords:** autonomy, personal development, personal growth, qualitative study, responsibility

## Abstract

Clinical experiences, helping relationships, and reflection are key factors for personal development for physicians. However, few studies have shown which experiences are important for personal growth and how medical residents specifically use their experiences for personal growth. The aim of this study was to identify from the medical residents’ perspective which clinical experiences contribute to their personal development. We employed a qualitative design, conducting semi-structured interviews with ten postgraduate year two medical residents at a Japanese teaching hospital. The interviews were transcribed in interview memos, anonymized, and subjected to reflective thematic analysis to generate themes relevant to personal and professional development. Successful clinical experiences with autonomy and responsibility in clinical management were shown to be essential points for personal development as a physician. Autonomy in this study was the attitude of making one’s own choices when managing patients. Responsibility was the obligation of the resident to take charge of a patient. Instructing junior trainees, appreciation received from patients, and approval granted by attending physicians reinforced their feelings of personal growth. The realization of what experiences and concepts influence medical residents’ personal growth and development will make their professional development more effective.

## 1. Introduction

Physicians need to have not only clinical knowledge and skills but also the ability to have appropriate communication with patients and other healthcare professionals, as well as professionalism. Physicians in training should naturally develop these competencies and should strive to do so. A study on personal growth in a medical faculty showed that experience, helping relationships, and reflection are crucial elements for personal development [1]. It was revealed in this study that personal growth includes orienting towards values, goals, and attainment targets; healthier behavior; improved relationships with others; an improved sense of self; and increased productivity, energy, and creativity [1]. These components are related to Kolb’s learning cycle [2] and represent a learning cycle facilitated by action, reflection, and revealing the double dialectic of experience and abstraction. These two dimensions define an overall learning space in which learning transactions take place between the individual and the environment. The dimensions of learning are multi-level and can develop learning and enhance individual development [3].

Research conducted with medical residents revealed distinctive experiences as triggers and catalysts for personal growth and development. These experiences were significant, evoking intense emotions in medical residents and challenging their values and sense of individuality. Recurring themes included managing critically ill or dying patients, receiving feedback, witnessing unprofessional behavior, experiencing personal problems, and increased responsibility as a clinical resident [4]. Factors that facilitate personal growth, including supportive relationships, reflection, and commitment to fundamental values, were also identified in this study. Supportive relationships allowed clinical residents to talk to people who understood and cared about their experiences [4]. This study also showed that fatigue, lack of personal time, and feeling overwhelmed at work were major obstacles to personal growth [4]. A quantitative study using a personal growth scale for medical residents revealed that factors independently associated with achieving high degrees of personal growth during residency training included agreeing that reflection is important during residency training, being male, being non-white, having a strong desire to develop personally and professionally, and feeling highly supported by one’s program director [5]. Independent predictors of scoring below the median on the personal growth scale included feeling emotionally isolated at work and noting that negative or disappointing experiences had been powerful [5].

The above-described preliminary investigations focused on catalysts for personal development within the realms of medical practice and residency as well as the broader spectrum of clinical experiences that either foster or hinder such developmental processes [1,4,5]. There was a study that identified the process of increasing intrinsic motivation among medical residents that may possibly be involved in personal development [6]. However, a comprehensive examination of the fundamental constituents intrinsic to the role of a physician remains conspicuously absent from the existing literature.

The aim of this study was to determine the fundamental constituents of the medical profession that underpin the clinical experiences responsible for driving individualized personal growth and development.

## 2. Methods

### 2.1. Study Design and Participants

We conducted a qualitative study [7] and analyzed data using an inductive analytical method with a focus on reflective thematic analysis [8,9,10,11] based on a social constructivism paradigm. The target participants were postgraduate year two medical residents who mainly handled inpatients at the training hospital. We selected study participants through convenience sampling, whereby we asked those who were capable of participating to take part in the study. We continued sampling and analysis until saturation was reached with no codes and themes emerging from the data [12,13]. This sampling was conducted from September 2014 to March 2015, and ten participants were finally recruited.

### 2.2. Context of Training Environment

#### 2.2.1. Postgraduate Medical Resident Training System in Japan

This study was conducted in a Japanese teaching hospital. Japan is considered to be an appropriate country for conducting this study because Japan has a developed healthcare system and is internationally certificated for the quality of medical education [14,15]. In Japan, postgraduate medical students have a unique clinical internship system in which they rotate through various departments for two years after graduation from medical school. During the first two years after graduation from medical school, Japanese clinical residents receive training not only in emergency medicine, internal medicine, and surgery but also pediatrics, obstetrics and gynecology, psychiatry, and rural/family medicine [16]. After completion of the two-year postgraduate clinical training, residents can continue their specialized training for basically three years in order to obtain a specialist qualification.

#### 2.2.2. Context of Teaching Hospital and Program Where This Study Was Conducted

This research was conducted in a public 550-bed teaching hospital that provides tertiary care with a large and active emergency center in Okinawa Prefecture in Japan. During the first two years for postgraduate medical students, most rotations include overnight on-call shifts for inpatients and outpatient clinics once a week in addition to handling inpatient care. In this hospital, the responsibility for managing inpatient care primarily rests with second-year postgraduate medical residents, who take the lead in proactive management. On the other hand, first-year postgraduate residents do not directly engage in patient-handling duties. They usually support the work of second-year postgraduate medical residents.

### 2.3. Interviews

We performed semi-structured interviews. Before the interviews started, the participants were asked to provide individual information (gender, age, and training programs). The interviewer (KT) then asked each participant questions according to the interview guide (Table 1). The interviewer listened to the answers to the questions and added specific questions during the interview exchange to further interpret the growth and development of the participant as a physician and to detail the process of the participant’s development. Each interview lasted for 20–30 min. KT, who was working as a fellow physician, interviewed all of the participants. He conducted the interviews in Japanese. This was due to KT’s superior comprehension of the research questions. KT was not involved in the management or assessment of the participants’ training.

### 2.4. Data Collection

Semi-structured interviews were conducted to explore each part participant’s perspectives and meaningful experiences to accelerate the rich and detailed exploration of the research questions [17]. All participants were briefed on the interviews and indicated their willingness to participate. Interviews were conducted using the interview guide shown in Table 1. All interviews were documented in interview memos.

### 2.5. Data Analysis

The qualitative data were analyzed using reflective thematic analysis [8,9,10,11] with a focus on two major questions, shown in Table 1. Reflective thematic analysis is a method for identifying, analyzing, and reporting patterns or themes within data. It minimally organizes the obtained qualitative data and describes them in rich detail [8,9,10,11].

We chose this method because of the straightforward nature of the analysis process. Furthermore, this method allowed for the integration of the social constructivist paradigm [18] with qualitative data analysis based on a relatively small sample of qualitative data. The analysis was first conducted in Japanese by the principal investigator (KT) and a co-researcher (HO; Haruo Obara) as an independent auditor interpreted all records and independently reviewed the process at each stage of the analysis. In addition, co-researchers (HO: Haruo Obara, TH, HO: Hiroko Ogawa, and FO) interpreted, confirmed, and finalized all codes and themes. All of the authors held discussions that led to a consensus regarding the results. During this process, new codes emerged and codes were modified from different positions (KT was a general practitioner; HO, Haruo Obara was an internist; TH was an internist and hospitalist; HO, Hiroko Ogawa was specialized in internal medicine and rural medicine; and FO was specialized in endocrinology). All of the authors eventually reaffirmed the codes and themes from this analysis process. Finally, the conceptual structures related to the growth and development of the medical residents from their perspective were identified.

### 2.6. Ethical Considerations

Ethical considerations for this study were first reviewed and approved by the Ethics Committee of Okinawa Chubu Hospital (Approval number: H26 Chu Rin Sho Dai 54 Gou). For further consideration, a dedicated private space was set up in the researchers’ facility to protect the psychological and environmental safety of the participants and to ensure their privacy. The participants were also assured that their willingness to participate or not would not affect the benefits they would receive or the clinical training this study would provide. Written informed consent was obtained from each participant. In addition, participants were provided with assurances regarding the confidentiality and anonymity of their records and the behavior observed during the interviews. They were also informed that participation was voluntary and that they could withdraw at any time. Finally, it was emphasized that the information obtained would only be used for research purposes.

## 3. Results

Ten medical residents in the teaching hospital participated in this study. Their ages ranged from 26 to 30 years. Five participants were women and five were men. The departments they aspired to work in were varied. Table 2 shows the self-identified characteristics of the participants.

### 3.1. Successful Experiences with Autonomy and Responsibility

As physicians who were just starting to build their careers, the medical residents felt they had grown as physicians when they were able to do things they could not do before. This was described as successful experiences from their own proactive medical practice with autonomy in decision-making and responsibility for patient care. Resident 6 placed significant emphasis on the ability to perform tasks in clinical practice. Furthermore, through the accumulation of clinical experiences, the resident observed a notable increase in the effectiveness of her work.

*(…as a physician, I experienced personal development) when tasks were executed efficiently and promptly. This realization became apparent during my rotations within the internal medicine department over the course of the year. I observed that tasks tended to be completed more swiftly during the final rotation. For instance, in the gastrointestinal department, the tasks are relatively straightforward and their completion becomes progressively expedited as one becomes more acclimated to the routine. As my experience accumulates, I find that my efficiency increases. Through interactions with attending doctors, I glean insights into what aspects require careful consideration and where my focus should be directed.*

(Resident 6: PGY-2, female, 26 years old)

#### 3.1.1. Autonomy in Decision-Making

It was also important that the clinical management of patients during admission was semi-independent. Autonomy in decision-making is a necessary factor for clinical management by a physician. Autonomy in this study was defined as the attitude of having one’s own choice in managing patients.

*When I excel during an attending physician’s ward round, (I experience a profound sense of personal growth as a physician). This occurs when I am able to assess patients’ conditions and formulate effective treatment plans appropriately. Subsequently, I engage in informed discussions about the patients, presenting my assessments and plans during the attending physician’s ward round. Additionally, I have to manage the inpatients… treating, and providing care to the patients until their discharge. This enhanced involvement enables me to contribute more actively to healthcare practices, (thereby fostering my ongoing personal development).*

(Resident 2: PGY-2, female, 26 years old)

The medical residents actively engaged in proactive clinical practice by directly communicating with patients, identifying issues, and independently finding solutions, thus fostering their own personal development through successful experiences.

*(After becoming a second-grade medical resident,) I have taken the initiative to engage in direct conversations with my patients, identifying novel issues and successfully addressing them on my own. (This active involvement in patient care has led to) a tangible sense of personal growth. To illustrate, I encountered a situation where I was able to intervene in untreated cases of diabetes, dyslipidemia, and hypertension in a patient who had been admitted for a stroke.*

(Resident 4: PGY-2, male, 26 years old)

#### 3.1.2. Responsibility for Patient Care

Responsibility for patient care was the next key element in the development process of medical residents. Responsibility in this study was defined as a feeling by residents that they must take charge of their patients.

*I felt a sense of responsibility when I performed surgery on a patient. I want to be a responsible surgeon. I wonder if my patient will develop a fever. The responsibility is far different from that when I was postgraduate year one. (I experience a sense of personal growth) within the realm of surgery when I witness my patients who have been operated on being discharged (from the hospital and) returning home. This sentiment is particularly pronounced in cases like appendicitis. I embraced a profound sense of responsibility during my surgical procedure for a patient. This experience ignited a strong aspiration within me to evolve into a responsible (surgeon). I have concerns about the possibility of my patients, (for whom I have performed procedures), developing a fever.*

(Resident 3: PGY-2, male, 26 years old)

### 3.2. Instructing Junior Trainees, Appreciation Received from Patients and Approval Granted by Attending Physicians

Clinical experiences of personal growth for residents included teaching junior trainees and receiving appreciation from patients as well as approval of their clinical management from their attending physicians.

#### 3.2.1. Instructing Junior Trainees

Medical residents articulated their personal growth and development through their capacity to instruct junior trainees, forming a distinctive facet of their developmental profile, and enabling them to tackle tasks that were previously beyond their reach.

*(Feeling of personal growth) is most pronounced for me during interactions with interns. If I find myself unable to effectively guide interns, I question whether my own personal growth is truly occurring.*

(Resident 5: PGY-2, male, 29 years old)

#### 3.2.2. Appreciation Received from Patients and Approval Granted by Attending Physicians

The appreciation received from patients and the approval granted by attending physicians for the clinical actions provided medical residents with a chance to experience their own personal growth as a physician.

*I derive a sense of satisfaction when patients or their families express gratitude, confirming the effectiveness (of the therapeutic action) I have taken.*

(Resident 3: PGY-2, male, 26 years old)

*I recognize my personal development when I am able to make judicious selections of antibiotics and fluids and when I can adeptly manage inpatients. This progression reaches its pinnacle (when the attending physician) not only concurs with my choices but also grants their approval.*

(Resident 7: PGY-2, female, 27 years old)

Clinical management, conducted semi-independently as a physician, involves the process of taking a history from a patient, performing a physical examination, assessing, diagnosing, and forming a management plan for patient care. This experience of clinical management enabled the medical residents to understand what they could not do and learn what to do next.

## 4. Discussion

The findings presented in this article were derived from a qualitative interpretation of medical residents. The results of this study showed some methods of personal development as a physician among medical residents. Residents were aware of their personal growth and development as physicians through successful clinical experience with autonomy in decision-making and responsibility for patient care. Autonomy and responsibility were found to be crucial concepts for physicians. Medical residents who were new to the medical profession might have found that autonomy and responsibility were deeply involved in their successful clinical experience of personal development.

The clinical experiences of the medical residents that were described as successful experiences included successful surgical interventions, efficient work, and finding and solving patient problems. In social cognitive theory, mastery experiences are linked to personal development through self-efficacy. Efficacy beliefs are comparable across cultures not only in their structure but also in their functional characteristics [19,20,21]. In the more individualistic American social system, self-efficacy beliefs regulate self-learning, and self-efficacy to master academic activities has been reported to be a positive predictor of academic motivation and academic achievement, taking into account past achievement [22,23,24].

According to previous research in the fields of psychology and ethics, autonomy and responsibility are closely related [25]. Individuals need to have authenticity, self-control, and moral responsibility for their actions in order to have autonomy [26]. Transitional experiences regarding personal achievement and status advancement were shown to be highly correlated with ego development, while transitional experiences focused on personal growth in relationship involvement were shown to be more highly correlated with intrinsic components and greater subjective well-being [25].

The results of this study revealed that the autonomy of the medical resident handling patient care was one of the key concepts. In self-determination theory, an important theory regarding autonomy, the psychological need for autonomy, along with competition and relationships, is considered to promote better functioning for growth and to be essential for social development and individual well-being [27]. This theory is concerned with human motivation and personality and, while using traditional methods, adopts an organismic metatheory that emphasizes the importance of developed human internal resources for personality development and the self-regulation of behavior [28] The main areas of this theory cover personal tendencies and innate psychological needs regarding the growth inherent in people as the basis for self-motivation and personality development, as well as factors that facilitate these processes [27]. For individuals to successfully exercise their own autonomy, they adopt their own community-based principles. They possess self-governance capacities, values developed by the subject, and the way they internalize these values in themselves is social [29]. Furthermore, the development of one’s sense of self-importance depends on the social conditions that foster this capacity [30]. Social structures may articulate the value concepts available to people to develop their personal principles and individual values. A person’s ability to incorporate their values into key decisions is determined by the opportunities that exist in the wider social structure and extended social context [31]. In a study exploring the process of intrinsic motivation among medical residents, an environment in which they could take ownership, such as on-call shifts or in an emergency room, triggered perceptions of intrinsic motivation in medical residents [6]. In terms of social and environmental context, external factors may influence residents’ autonomy in decision-making.

Responsibility for patient care is also key to increasing intrinsic motivation [32]. In investigations regarding physicians’ core beliefs and attitudes, these were seldom fully articulated and sometimes organized into a personal philosophy about life [33]. These beliefs and attitudes explain why things happen as they do, define right and wrong, and characterize the nature of one’s responsibility toward others [34]. One possible explanation for why medical residents in this study seemed to have responsibility may be that they played an important role in inpatient care after becoming second-year postgraduate medical residents. Culturally, the Japanese are known to have a strong collective consciousness, which is often referred to as collectivism, and are often perceived as not being adept at individual decision-making [35,36]. Second-year residents might have primarily worked with inpatients within the single-*shujii* system [37], which is a unique healthcare system in Japan. In this system, one doctor is in charge of a patient. This is different from the Western healthcare system (multiple-*shujii* system, known as a team system). Within this culture of collectivism, medical residents might have experienced a heightened sense of autonomy and responsibility, given that their work required them to make decisions independently. On the other hand, physicians’ responsibility was noted as stress during postgraduate residency training [38]. Careful and supportive training should be conducted because this dysfunctional belief might hinder the personal growth of physicians.

Additionally, there were results showing that appreciation received from patients and approval granted by attending physicians made the medical residents notice their personal growth. Encouragement from others has been reported as a factor that strengthens self-efficacy, and the recognition that was felt to have grown from this clinical training may have been obtained through self-efficacy [39,40]. 

This article makes a novel point: autonomy and responsibility are the foundational components inherent to the medical profession that can drive personal growth and development as a physician. Autonomy in decision-making and the responsibility for patient care emerge as pivotal concepts among medical residents engaged in clinical practice. As a result, clinical educators are positioned to bolster residents’ clinical proficiency by emphasizing and nurturing these fundamental principles.

### 4.1. Implications and Future Research 

Knowing and interpreting what experiences and concepts exist in actual clinical practice and how these experiences and concepts influence medical residents’ personal growth and development will make their personal development more effective. Clinical training for medical residents will be better if the clinical experience with autonomy and responsibility is successfully incorporated within the clinical education setting.

Future research is needed to determine when medical residents’ autonomy in decision-making and responsibility for patient care emerges. Significant past experiences, the ideal image and the values of a physician, their influence on autonomy and responsibility, and the process of establishing autonomy and responsibility should be investigated. Research showing the relationship between personal growth and self-efficacy could be significant, as self-efficacy might be a core concept in personal growth and the development of medical residents. Furthermore, responsibility for patient care is strongly related to the concept of patient care ownership, which is an important component of medical professionalism [37,41]. The responsibility and patient care ownership inherent in the medical professional development process should also be elucidated.

### 4.2. Limitations

The findings of this study should be considered within the methodological, cultural, and environmental limitations. First, some of the clinical experiences and growth/development processes that medical residents perceive as their personal growth/development as a physician were presented. These findings were obtained from a small number of participants. Second, regarding recruitment methods, convenience sampling was used, as it was difficult to recruit widely and seek free participation in the study. This may have inherent selection bias, as the researchers partially selected the study participants. Finally, participants were selected from a single East Asian country, Japan. Japan has its own traditional culture, and it is possible that the descriptions of the clinical residents may have been influenced by their cultural background.

## 5. Conclusions

Medical residents are aware of their personal growth and development as physicians through successful clinical experience with autonomy in decision-making and responsibility for patient care. The appreciation received from patients and the approval granted by attending physicians for the clinical actions also provided medical residents with their own personal growth as physicians.

## Figures and Tables

**Table 1:** Interview guide.

1	When did you experience personal growth and development in your role as a physician?
2	What kind of clinical experience has progressed your personal development as a physician?

**Table 2:** Demographics of participants.

Participant	Self-Identified Characteristics		
	Gender	Age (years)	Aspiring Specialty as a Doctor
#1	Male	26	Emergency room doctor
#2	Female	26	Internal medicine doctor
#3	Male	26	Surgeon
#4	Male	26	General practitioner
#5	Male	29	General practitioner
#6	Female	26	Internal medicine doctor
#7	Female	27	Internal medicine doctor
#8	Male	26	Surgeon
#9	Female	30	General practitioner
#10	Female	26	Emergency room doctor

All participants were postgraduate year two medical residents and they were all Japanese.

## Data Availability

The data presented in this study are available on request from the corresponding author.
